# Leiomyosarcoma of the Prostate: Case Report and Review of 54 Previously Published Cases

**DOI:** 10.1155/2008/458709

**Published:** 2008-11-18

**Authors:** Gerasimos P. Vandoros, Theodoros Manolidis, Michalis V. Karamouzis, Maria Gkermpesi, Maria Lambropoulou, Athanasios G. Papatsoris, Ioannis Zachos, Panagiotis A. Konstantinopoulos

**Affiliations:** ^1^Department of Pathology, Aeghion General Hospital, 25100 Aeghion, Greece; ^2^Department of Biological Chemistry, Medical School, University of Athens, 115 27 Athens, Greece; ^3^Division of Hematology/Oncology, University of Pittsburgh, Pittsburgh, PA 15232, USA; ^4^Department of Histology/Embryology, Democritus University of Thrace, 68 100 Alexandroupolis, Greece; ^5^Division of Hematology and Oncology, Harvard Medical School, Beth Israel Deaconess Medical Center, Boston, MA 02215, USA

## Abstract

Prostate leiomyosarcoma is an extremely rare and highly aggressive neoplasm that accounts for less than 0.1% of primary prostate malignancies. We present a patient with primary leiomyosarcoma of the prostate and review 54 cases reported in the literature to discuss the clinical, diagnostic and therapeutic aspects of this uncommon tumor. Median survival was estimated at 17 months (95% C.I. 20.7–43.7 months) and the 1-, 3-, and 5-year actuarial survival rates were 68%, 34%, and 26%, respectively. The only factors predictive of long-term survival were negative surgical margins and absence of metastatic disease at presentation. A multidisciplinary approach is necessary for appropriate management of this dire entity.

## 1. INTRODUCTION

 Prostate leiomyosarcoma is an extremely rare neoplasm that accounts for less than 0.1% of primary prostate malignancies [1]. It is the most common primary sarcoma of the prostate in adults and comprises 38% to 52% of primary prostatic sarcomas [2]. We present a patient with primary leiomyosarcoma of the prostate and review 54 cases reported in the literature to discuss the clinical, diagnostic, and therapeutic aspects of this uncommon tumor.

## 2. CASE REPORT

An 80-year-old man presented with frequent micturition, dysuria, poor urinary stream, and nocturia of approximately 12-month duration. He reported no hematuria or perineal pain and denied any constitutional symptoms. There was no family history of genitourinary cancer. He was a heavy smoker, drank alcohol socially, and reported no exposure to hazardous chemicals. Rectal examination revealed a firm nodular mass, 3 to 4 cm in diameter, involving the left lobe of the prostate and extending to the edge of the gland. The right prostatic lobe was diffusely firm. There was no palpable lymphadenopathy, and the rest of his physical examination was unremarkable. Prostate specific antigen (PSA) at presentation was 2.7 ng/mL, and his creatinine was normal. His last PSA, obtained 3 years earlier by his primary care physician as part of routine annual physical examination, was the same.

**Figure 1:**
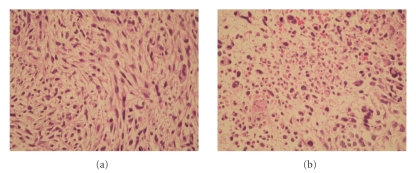
Leiomyosarcoma composed of a dominant population of neoplastic spindle cells: (a) intermingled with giant neoplastic cells and multifocal necrosis (b).

**Figure 2:**
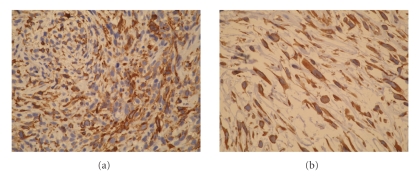
Immunohistochemistry demonstrates that tumor cells express smooth muscle actin (a) and vimentin (b).

Patient underwent transurethral resection of the prostate (TURP), and pathology revealed a dominant population of neoplastic spindle cells intermingled with giant neoplastic cells and multifocal necrosis that involved almost the entire tumor (Figures 1(a), 1(b)). Immunohistochemistry confirmed the diagnosis of leiomyosarcoma of the prostate. Specifically, tumor cells expressed smooth muscle actin (Figure 2(a)), vimentin (Figure 2(b)), and CD44, while they exhibited no staining for S-100, cytokeratins, and CD117 (c-KIT).

Computed tomography (CT) of the abdomen demonstrated two hypodense liver lesions, and CT scan of the chest showed multiple pulmonary nodules and mediastinal and left hilar lymphadenopathy, all considered suspicious for metastatic disease. CT of the brain and bone scan was negative for metastatic disease.

Patient denied any intervention and was treated symptomatically. Three months later, he presented with urinary retention and acute renal failure. A permanent urinary catheter was placed, and a palliative external beam radiotherapy was recommended. Patient denied any treatment and was discharged home with hospice. He died 2 weeks later.

## 3. RESULTS

The information for the 54 cases included in this review was compiled using the PubMed and Medline databases for articles published in the last 20 years until March 1, 2008 (Table 1). The search terms used were prostate, sarcoma, leiomyosarcoma, and malignancy. Only articles published in English were considered.

The median age of the 54-patient cohort included in this review was 63.8 years (ranging from 40 to 80). The most common presenting manifestations (among 38 patients for whom clinical data regarding presenting symptoms were available) included obstructive urinary symptoms in 89.4% and perineal or rectal pain in 25.6% of the patients. Less frequent manifestations were burning on ejaculation and hematuria, both presented as initial symptoms in only 5.2% of the patients (Table 2). The diagnosis was obtained by ultrasound-guided transrectal needle biopsy or TURP in the majority of patients, whereas transperineal biopsy, CT-guided biopsy, or suprapubic prostatectomy was only rarely necessary.

A sizeable proportion of patients (23.5%) had metastatic disease at the time of diagnosis. Lungs were the most common sites of metastatic disease accounting for 17.6% of the cases, followed by liver (11.7%), and bone (5.8%) (Table 2). Only two patients had metastatic disease in the brain (3.6%). 61.7% of the patients, included in this review, underwent surgical resection: 35.5% received external beam radiation therapy, and 41.1% were treated with adjuvant or neoadjuvant chemotherapy.

**Figure 3:**
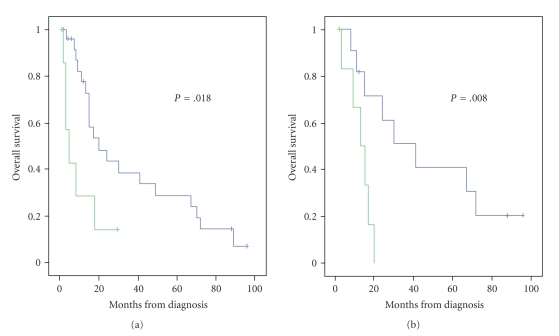
(a): patients with metastatic disease at presentation had worse overall survival than those with no metastatic disease (median survival for 5 months versus 20 months, resp., *P* = .018). (b): patients with microscopic or gross residual disease after surgery had worse overall survival than those with microscopically negative margins after surgery (median survival for 13 months versus 41 months, resp., *P* = .008).

Among 55 patients (including our patient), clinical outcome data were available for 34 patients. Median survival was 17 months (95% CI 20.7–43.7 months) and the 1-, 3-, and 5-year a ctuarial survival rates were 68%, 34%, and 26%, respectively. Our analysis in this 34-patient cohort demonstrated that the only factors predictive of long-term survival were absent from metastatic disease at presentation and negative surgical margins (Figures 3(a) and 3(b), resp.). Specifically, patients with metastatic disease at presentation had worse overall survival than those with no metastatic disease (median survival for 5 months versus 20 months, resp., *P* = .018), and patients with microscopic or gross residual disease after surgery had worse overall survival than those with microscopically negative margins after surgery (median survival for 13 months versus 41 months, resp., *P* = .008).

## 4. DISCUSSION

Primary prostate sarcomas arise from nonepithelial mesenchymal components of the prostate stroma and account for less than 0.1% of primary prostate tumors [1]. Leiomyosarcoma is the most common histological type in adults (38% to 52% of primary prostatic sarcomas), while rhabdomyosarcoma is the most common in pediatric patients [1, 2].

Leiomyosarcoma most commonly presents with signs and symptoms of urinary obstruction (frequency, urgency, and nocturia), as well as hematuria, perineal and/or rectal pain, constipation, burning on ejaculation, and constitutional symptoms such as weight loss [2–7]. In the 54-patient cohort, obstructive urinary symptoms and perineal or rectal pain were the most common presenting manifestations.

Physical examination reveals nonspecific enlargement of the prostate, while serum PSA is typically within normal limits [2, 3, 7]. Diagnosis is accomplished by ultrasound-guided transrectal needle biopsy or TURP in most patients and less commonly by transperineal biopsy, CT-guided biopsy, or suprapubic prostatectomy [2]. Lesions typically range between 2 and 31 cm and are frequently very infiltrative with focal areas of hemorrhage, necrosis, and/or cystic degeneration [1, 8].

The majority of leiomyosarcomas are high-grade hypercellular lesions composed of intersecting bundles of eosinophilic spindle-shaped cells with increased mitotic activity and moderate to severe nuclear atypia [8]. High-grade leiomyosarcomas typically exhibit prominent necrosis and cystic degeneration. Low-grade leiomyosarcomas, with moderate atypia, scattered mitoses, and a focally infiltrative growth pattern around benign prostate glands, are very rare [8]. Neoplastic cells commonly express vimentin, smooth muscle actin, and desmin, while cytokeratin expression is observed only in approximately 25% of the cases [3]. Some leiomyosarcomas express progesterone receptor, whereas S-100 and CD117 are negative in all tumors [9]. Cytogenetic analysis of primary prostatic leiomyosarcomas reveals clonal chromosomal rearrangements involving chromosomes 2, 3, 9, 11, and 19 [10].

The local extent of prostatic leiomyosarcoma is determined by CT or MRI scans, which provide clear delineation of the tumors from surrounding normal tissues and are important in assessing whether they are surgically resectable. A significant proportion of these neoplasms presents with metastatic disease. In the 54-patient cohort, lungs were the most common sites of metastatic spread followed by liver and bone. In that regard, chest CT constitutes an important component of the metastatic evaluation of prostatic leiomyosarcomas. Since brain metastases are uncommon, imaging of the brain should not be performed routinely, unless there is high-clinical suspicion [2, 3].

Multimodality treatment combinations including surgery, pre- or postoperative radiation therapy, and neoadjuvant or adjuvant chemotherapy have been used in the management of leiomyosarcomas of the prostate, but there are no standard treatment recommendations [2–4, 7, 11]. Operable tumors are treated with surgery, which may be followed by radiation therapy and/or adjuvant chemotherapy, particularly in patients with positive margins or nodes [11]. Patients with bulky disease may be treated with neoadjuvant (preoperative) chemotherapy with or without radiotherapy followed by an attempt for surgical resection. In patients with inoperable or disseminated disease, systemic chemotherapy may induce clinical responses, but these rarely translate into sustained remission [2, 3, 12]. Patients who develop isolated pulmonary metastatic disease after complete resection of the primary tumor may be offered the option of surgical resection, as this can be sometimes associated with long-term survival [13].

Surgeries with curative intent include radical retropubic prostatectomy, radical cystoprostatectomy, suprapubic prostatectomy, and pelvic exenteration [2–4, 11]. Various chemotherapy regimens have been used in this disease, but most patients receive anthracycline (doxorubicin or epirubicin)-based combinations with alkylating agents (cyclophosphamide, ifosfamide, or dacarbazine) and/or vinca alkaloids (vinblastine or vincristine) [2, 14–16]. Platinum-based combinations have also been used with mixed results [2, 17].

The clinical outcome of patients with prostate leiomyosarcoma is poor. The 17-month median survival estimated in our retrospective analysis renders prostate leiomyosarcoma as one of the most aggressive prostate malignancies, similar to other histologic subtypes of prostate soft-tissue sarcomas, more aggressive than prostate adenocarcinoma, albeit somewhat less aggressive than prostate carcinosarcoma, which is associated with an actuarial risk of death of 20% within 1 year of diagnosis and frequent widespread metastases to bones, liver, and lungs. When compared to other urologic leiomyosarcomas, prostate leiomyosarcomas are associated with significantly worse survival than renal and bladder leiomyosarcomas [18, 19]. Our retrospective analysis revealed that the presence of metastatic disease at presentation and the presence of positive surgical margins are associated with adverse outcome. This finding is in agreement with the study published by Sexton et al. [2] although their analysis involved all prostate sarcomas (all histologic types of prostate sarcomas grouped together) and did not specifically examine prostate leiomyosarcomas.

In conclusion, leiomyosarcoma of the prostate is a rare neoplasm that usually presents with metastatic disease and typically follows an aggressive course. A multidisciplinary approach that includes urology, radiation, and medical oncology consultations should be employed for appropriate management of this devastating malignancy.

## Figures and Tables

**Table 1:** 54 cases of primary prostate leiomyosarcoma included in this review.

Study	Year	Patients
Dotan et al. [4]	2006	8
Talapatra et al. [7]	2006	1
Sexton et al. [2]	2001	12
Cheville et al. [3]	1995	23
Dundore et al. [5]	1995	5
Russo et al. [6]	1993	1
Ahlering et al. [11]	1988	4

**Table 2:** Clinical characteristics of patients with prostate leiomyosarcoma (retrospective review of 55 patients including this case).

Clinical characteristics		Percent of patients^++^
Presentation	Obstructive symptoms	89.4%
	Perineal/rectal pain	25.6%
	Hematuria	5.2%
	Painful ejaculation	5.2%

Metastatic disease at presentation	All sites	23.5%
	Lung	17.6%
	Liver	11.7%
	Bone	5.8%

Therapy	Surgery	61.7%
	Radiation	35.3%
	Chemotherapy	41.1%

Survival	1 year	68%
	3 years	34%
	5 years	26%

^++^Percentage is based on the patients for whom clinical data were available in each case.
